# Pantoea graminicola sp. nov., a Gram-negative bacterium isolated from sweet corn (Zea mays L.) in the USA

**DOI:** 10.1099/ijsem.0.006952

**Published:** 2025-11-03

**Authors:** Larissa C. Ferreira, Vitor A. S. de Moura, Mousami Poudel, Kiersten R. Fullem, Jeffrey B. Jones, Katia V. Xavier

**Affiliations:** 1 Department of Plant Pathology, Everglades Research and Education Center, University of Florida, Belle Glade, FL 33430, USA; 2 Department of Plant Pathology, University of Florida, Gainesville, FL 32611, USA

**Keywords:** Everglades Agricultural Area, Florida, maize, phytopathology

## Abstract

In 2024, bacterial strains KXB24, KXB25 and KXB45 were isolated from sweet corn (*Zea mays* L.) plants exhibiting foliar chlorosis and streaking, typical symptoms of bacterial infection, in the Everglades Agricultural Area in Florida, USA. A polyphasic taxonomic study was conducted on these strains. Phylogenetic analysis of 16S rRNA gene sequences, whole-genome comparisons and biochemical characterization confirmed that the isolates belong to the *Pantoea* genus. The Biolog Gen III MicroPlate system identified substrate utilization profiles that were unique to these strains, distinguishing them from closely related *Pantoea* species. The strain KXB24 showed positive utilization of d-serine, γ-amino-butyric acid, citric acid and other compounds. On nutrient agar, KXB24, colonies are circular, smooth, convex and yellow and do not produce fluorescent pigment on King’s medium B. The DNA G+C content of KXB24 was determined to be 57.0 mol%. Based on phenotypic distinctions, phylogenomics evidence, average nucleotide identity values below 94.26% and digital DNA–DNA hybridization values below 57.9% when compared to type strains of recognized *Pantoea* species, we propose *Pantoea graminicola* sp. nov., with KXB24^T^ (=NCPPB 4802^T^=LMG 33887^T^) as the type strain. This discovery enriches the current understanding of *Pantoea* species and calls for further research into the potential role of *P. graminicola* in sweet corn disease aetiology and its broader agricultural impacts.

## Introduction

Maize (*Zea mays* L.), commonly known as corn, is a vital crop worldwide, with the USA being the leading producer and consumer. While maize cultivation in the USA is largely concentrated in the Midwest [1], Florida occupies a unique position, especially in Palm Beach and Miami-Dade Counties, which are among the top producers of sweet corn in the country [2,3]. The Everglades Agricultural Area (EAA), located in Palm Beach County, plays a key role in sweet corn production. This region is characterized by a unique agricultural system, where sweet corn is grown during the winter in rotation with sugarcane (a semi-perennial crop) and rice (a summer crop) [4,5]. In January 2024, a previously unreported bacterial disease emerged in sweet corn fields within the EAA, characterized by chlorosis and necrotic streaks in the foliage, similar to symptoms of bacterial leaf streak (*Xanthomonas vasicola* pv. *vasculorum*). The disease incidence was nearly 100% across 32 ha of sweet corn, raising concerns about potential yield losses in this critical production area. In response to this outbreak, bacterial strains were isolated from symptomatic corn plants. While these isolates were found to be associated with diseased plants, their exact role in the disease remains undetermined.

The isolated strains were identified as members of the genus *Pantoea* based on 16S rRNA phylogenetic analysis [6]. *Pantoea* species are versatile bacteria within the family *Erwiniaceae*, known for their diverse roles in various environments, including soil, water, plants and animal systems [7]. Species within this genus are known to infect a broad range of plants, including both monocots and dicots. For instance, *Pantoea ananatis* has been documented as a pathogen of crops such as rice, onions and corn [8], while novel *Pantoea* species have been discovered infecting wheat in the USA [9]. Genome-based species delineation has proven crucial in expanding our understanding of *Pantoea*, revealing 49 distinct species within the genus [10,11].

To elucidate the taxonomic identity of the bacterial strains associated with this outbreak, we employed a polyphasic approach in this study. By combining phylogenetic analysis based on core gene sequences, whole-genome comparisons and biochemical profiling, we aim to clarify the status of three strains isolated from infected sweet corn plants (KXB24^T^, KXB25 and KXB45). Based on the findings from our integrative analysis, these strains represent a novel species within the genus *Pantoea*, for which we propose the name *Pantoea graminicola* sp. nov.

## Methods

### Sample collection and bacterial isolation

Symptomatic leaf tissue was collected randomly from corn plants across four separate locations within the 80-acre (32 ha) sweet corn field in Palm Beach County on 16 January 2024. This sampling procedure was repeated on 6 February 2024, from an additional four locations. Leaf samples were subsequently examined under a light microscope for the presence of bacterial streaming. Samples were surface-sterilized as follows: small sections from the margins of symptomatic tissue (avoiding necrotic centres) were excised using sterile scalpels and forceps. The leaf sections (~1 cm²) were placed into cassettes and submerged in 70% ethanol for 30 s, followed by immersion in 1.2% sodium hypochlorite solution for 6 min. The cassettes were then rinsed three times, each time in a different sterile beaker containing sterile distilled water. After surface sterilization, the disinfested leaf sections were placed in a 2 ml tube containing 300 µl of autoclaved distilled water using sterile tweezers. The leaf sections were then ground using a plastic mini pestle attached to a drill. The resulting bacterial suspension was streaked onto nutrient agar (NA, Difco, USA) plates using the same mini pestle and incubated at 28 °C in the dark for 24–48 h. A single, typical colony from each plate was then re-streaked onto a fresh NA plate. A total of 17 isolates were obtained. For long-term storage, a single colony from a pure culture plate was transferred to Luria–Bertani broth (Difco, USA), incubated overnight at 28 °C with shaking (180 r.p.m.) and then mixed at a 1 : 1 ratio with 60% (v/v) glycerol solution before storing at −80 °C. Preliminary 16S rRNA gene sequencing identified the majority of these isolates as belonging to the genus *Pantoea*, while a few were affiliated with other genera. Three representative *Pantoea* strains (KXB24, KXB25 and KXB45) were selected for detailed characterization and further analysis.

### Whole-genome sequencing and 16S rRNA sequence analysis

Genomic DNA was extracted from bacterial strains KXB24, KXB25 and KXB45 using a SYNERGY 2.0 DNA Extraction Kit (OPS diagnostics, USA) with minor modifications. Total DNA obtained was eluted in 50 µl of Monarch^®^ DNA Elution Buffer (New England Biolabs, USA). Genomic DNA was sequenced at SeqCenter (Pittsburgh, PA, USA) using an Illumina NovaSeq X Plus sequencer. Following sequencing, demultiplexing, quality control and adapter trimming were performed using bcl-convert1 (v4.2.4). The genome assembly and annotation were conducted in Bacterial and Viral Bioinformatics Resource Center (BV-BRC) [12] using Comprehensive Genome Analysis Service. The reads were trimmed using Trim Galore v0.6.5dev, assembled using Unicycler v0.4.8, corrected using Pilon v1.23 and quality-assessed using QUAST v5.2.0 and CheckM [13,16].

A database comprising 16S rRNA sequences from the type strains of all validly published *Pantoea* species at the time of analysis (*n*=20) was constructed using data obtained from the List of Prokaryotic names with Standing in Nomenclature. In addition, a search of the Genome Taxonomy Database (GTDB) identified one additional strain sharing >95% ANI with our isolates, which was subsequently included in the analysis. These type strains are listed in Table 1. The sequences were aligned using MAFFT v.7 [17]. Phylogenetic reconstruction was conducted using the maximum likelihood method in IQ-TREE with 1,000 bootstrap replicates. The ‘auto’ option in IQ-TREE was used to automatically select the optimal evolutionary model for tree construction [18]. The resulting phylogenetic tree was visualized using the iTOL [19].

**Table 1.** Comparison of strain KXB24^T^ with type strains of all recognized *Pantoea* species in 16S rRNA sequence identity, average nucleotide identity (ANI) and digital DNA–DNA hybridization (dDDH) values. All values are expressed as percentages

Strain	16S rRNA sequence identity	ANI	dDDH
KXB25 (GCF_048452525.1)	100	100	100
KXB45 (GCF_048452585.1)	100	99.35	96.5
*Pantoea* sp003236715 ARC607 (GCF_003236715.1)	100	99.33	96.4
*Pantoea brenneri* LMG 5343^T^ (GCF_029505375.1)	99.67	87.62	34.8
*Pantoea conspicua* LMG 24534^T^ (GCF_002095315.1)	99.55	83.91	28
*Pantoea deleyi* LMG 24200^T^ (GCF_006494415.1)	99.42	94.26	57.9
*Pantoea anthophila* LMG 2558^T^ (GCF_006494375.1)	99.33	86.25	32.1
*Pantoea vagans* LMG 24199^T^ (GCF_004792415.1)	99.03	87.31	34.1
*Pantoea ananatis* LMG 2665^T^ (GCF_000661975.1)	99	78.47	22.4
*Pantoea allii* LMG 24248^T^ (GCF_002307475.1)	98.96	78.42	22.3
*Pantoea agglomerans* NBRC 102470^T^ (GCF_001598475.1)	98.66	87.62	34.7
*Pantoea eucalypti* LMG 24197^T^ (GCF_009646115.1)	98.61	86.53	32.6
*Pantoea stewartii* LMG 2715^T^ (GCF_045159535.1)	98.07	78.63	22.6
*Pantoea septica* LMG 5345^T^ (GCF_002095575.1)	97.54	79.65	23.2
Pantoea coffeiphila* 1480/Ca04^T^ (GCF_016909495.1)	97.4	74.99	20.5
*Pantoea wallisii* LMG 26277^T^ (GCF_002095485.1)	97.14	78.32	22.1
*Pantoea dispersa* DSM 30073^T^ (GCF_014155765.1)	97.02	78.75	22.4
*Pantoea rodasii* DSM 26611^T^ (GCF_002811195.1)	96.69	77.32	21.3
*Pantoea piersonii* IIIF1SW-P2^T^ (GCF_003612015.1)	96.67	79.22	22.9
*Pantoea rwandensis* LMG 26275^T^ (GCF_002095475.1)	96.58	76.64	20.9
*Pantoea eucrina* LMG 5346^T^ (GCF_002095385.1)	96.5	77.81	21.8
*Pantoea cypripedii* LMG 2657^T^ (GCF_002095535.1)	96.43	77.87	21.8
*Pantoea leporis* R^T^ (GCF_028048155.1)	96.39	77.85	21.6

*Pantoea coffeiphila strain 1480 used for genomic analyses, and type strain Ca04T used for 16S rRNA identity.

### Phylogenomic analysis

Genomes of the type strains listed in Table 1 were downloaded from GenBank and included in our in-house *Pantoea* genome database. The only exception was *P. coffeiphila*, for which strain 1480 was used in the genomic analyses due to the unavailability of genomic data for the type strain Ca04ᵀ. These genomes were used for phylogenomic analysis based on 1,000 single-copy core genes, as well as for calculating ANI and performing Genome-to-Genome Distance Calculator digital DNA-DNA hibridization (dDDH) comparisons. ANI values were calculated using the ANIb method on the JSpeciesWS server (jspecies.ribohost.com/jspeciesws/#Home), while dDDH values were determined using the Genome-to-Genome Distance Calculator (GGDC 3.0; ggdc.dsmz.de/ggdc.php) with the recommended formula 2.

A phylogenomic tree was constructed using the BV-BRC Phylogenetic Tree Building service (https://www.bv-brc.org/app/PhylogeneticTree). With this platform, 1,000 conserved genes were selected across the genomes, and RAxML was used for tree construction with 100 bootstrap replicates to assess robustness. The Type (Strain) Genome Server (TYGS; tygs.dsmz.de/) was also used, which automates the construction of species trees based on genome data. For this method, a phylogenetic tree was inferred with FastME 2.1.6.1 [20] from the Genome blast Distance Phylogeny (GBDP) approach under the algorithm 'coverage' and distance formula d5 [21].

### Analysis of type III secretion system

The search for type III secretion system (T3SS) proteins was performed using the tblastn algorithm against the genomes in our *Pantoea* spp. database (Table 1). Twelve amino acid sequences from three T3SS systems in *P. stewartii* subsp. *stewartii* DC283 were used as queries: HrcC, HrcN, HrcU and HrcV from the PSI-1 (Hrc 1) system; PsaC, PsaN, PsaU and PsaV from the PSI-2 (SPI-1) system; and SsaC, SsaN, SsaU and SsaV from the PSI-3 (SPI-2) system [22,23]. These amino acid sequences were used as they have been identified as core components of T3SS in *Pantoea* [22]. Hits with an E-value lower than 10e−20 were considered a positive match and retained for further analysis.

### Physiological and carbon utilization profiling

The phenotypic profiling of strains KXB24^T^, KXB25 and KXB45 was conducted using the Biolog Gen III MicroPlate system (S5). Each bacterial strain was cultured overnight on Biolog Universal Growth Agar (Biolog Inc., USA), and a small amount of bacterial growth was suspended in Biolog inoculation fluid 1 F-A (Biolog Inc., USA) to reach the turbidity level recommended. For each strain, 100 µl of the bacterial suspension was added to each well of the MicroPlate. The plates were incubated at 28 °C for 24 h, after which reactions in individual wells were recorded and compared to the Biolog database [14]. Strain identification was determined based on Similarity Index and Distance values. For comparative analysis, reference strains *P. agglomerans* CGMCC1.843^T^ [24], *P. deleyi* LMG 24200^T^ [25], *P. vagans* DSM 23078^T^, *P. allii* DSM 25133^T^ and *P. ananatis* ATCC 19321^T^ [26] were included alongside the three novel strains.

### MALDI-TOF MS

A pure culture of the bacterial strain KXB24^T^ was grown overnight on NA plates and sent to Charles River Laboratories (Newark, DE, USA) for analysis using MALDI-TOF MS. The resulting peptide mass spectra were compared with reference spectra in the Bruker MALDI Biotyper Sirius with the MBT Compass HT software (v. 5.1) and Charles River MALDI-TOF MS libraries (v. 25) for species identification.

### Pathogenicity and hypersensitivity response tests on maize, rice, tomato and tobacco

For all pathogenicity and hypersensitive response (HR) tests, the same bacterial suspension was used. Overnight cultures of KXB24^T^, KXB25 and KXB45 grown on NA media were suspended in a 10 mM MgSO₄ solution and adjusted to a concentration of ~10^8^ to 10^9^ c.f.u. ml^−1^. Sweet corn hybrids (CN361E, GSS1170 and BSS1075), field corn hybrid (DKC69-16) and criollo maize at the V3 growth stage were inoculated with the suspensions using multiple methods: spraying with the bacterial suspension, subcuticular injection with a needleless syringe and mild abrasion using a brush, carborundum or multi-needle. Control plants were mock-inoculated with 10 mM MgSO₄ without bacteria. After inoculation, plants were enclosed in plastic bags for 48 h to maintain high humidity (RH >98%), followed by 12 days in the greenhouse, totalling 14 days of observation after inoculation. This pathogenicity test was repeated 15 times. In some replicates of this experiment, plants were pre-treated by placing them in an intermittent mist chamber for 24 h prior to inoculation. Additionally, in some replicates, plants were incubated in a humidity chamber for 72 h after inoculation.

Similarly, 3-week-old rice plants (cultivars Diamond and Jewel) were inoculated by spraying the bacterial suspension, incubated in a humidity chamber for 48 h and kept in the greenhouse for a total of 14 days after inoculation. This experiment was conducted twice. Both maize and rice plants were assessed for symptoms at 7 and 14 days post-inoculation. Re-isolations were performed for both maize and rice at 14 days post-inoculation, following the previously described protocol, to confirm the presence of the inoculated strains. PCR amplification and sequencing of the 16S rRNA gene were carried out to confirm the identity of the re-isolated strains.

For the HR tests, tomato and tobacco leaves were injected with the bacterial suspension, and HR was assessed within 48 h of inoculation. This experiment was conducted twice.

### Transmission electron microscopy

Transmission electron microscopy of strain KXB24^T^ was performed at the University of Florida’s Interdisciplinary Center for Biotechnology Research to determine cell shape, size and flagellar arrangement.

### Comparison of genomes to GTDB

To determine whether any other bacterial isolates belonging to *P. graminicola* sp. nov. had been sequenced and published online, the genomes of isolates KXB24^T^, KXB25 and KXB45 were compared to GTDB using the software toolkit GTDB-tk with default parameters [27,28]. The program compares user-uploaded genomes to those uploaded to NCBI databases using whole-genome ANI and clustering to assign them taxonomic classifications.

## Results and discussion

### 16S rRNA phylogeny and sequence analysis

Strains KXB24^T^, KXB25 and KXB45 had identical 16S rRNA gene sequences. Phylogenetic analysis based on the 16S rRNA gene revealed that these strains formed a distinct clade that was sister to the *P. brenneri* lineage, supporting their status as a separate taxon (Fig. S1, available in the online Supplementary Material). This clade also includes strain ARC607, an isolate from rice (*Oryza sativa*) seeds in Benin, which represents the *Pantoea* sp003236715 lineage proposed by Crosby *et al*. [11] and shares 100% 16S rRNA gene identity with KXB24ᵀ. Among recognized type strains, KXB24^T^ showed the highest 16S rRNA gene sequence similarity (99.67%) with *P. brenneri* LMG 5343^T^ (Table 1). Sequence identities between our strains and type strains of *P. deleyi*, *P. conspicua*, *P. ananatis*, *P. allii*, *P. vagans*, *P. agglomerans*, *P. eucalypti* and *P. anthophila* ranged from 98.61% to 99.55% (Table 1).

### Genomic analysis

The whole-genome sequencing of strains KXB24^T^, KXB25 and KXB45 revealed consistent features across all strains (Table 2). Each genome exhibited 100% completeness and 0.2% contamination. The total number of contigs ranged from 25 to 29, with the largest contig in strain KXB45 reaching 1,286,269 bp. Total genome lengths varied slightly between 4,478,625 and 4,588,430 bp. Genome assembly quality, reflected by N50 and L50 values, showed that strain KXB45 had the highest N50 (537,076 bp) and lowest L50 (3), indicating a more contiguous assembly. Additionally, coarse and fine consistency values were nearly identical among the strains, demonstrating reliable genome annotations (Table 2).

**Table 2.** Genomic features of *Pantoea* strains KXB24^T^, KXB25 and KXB45 and their genome assembly statistics

Genome features	KXB24^T^	KXB25	KXB45
# contigs	29	26	25
# contigs (>=50,000 bp)	13	13	11
Largest contig	818,903	785,428	1,286,269
Total length	4,588,430	4,587,957	4,471,625
N50	474,595	488,243	537,076
N90	137,951	137,951	226,551
L50	4	4	3
L90	10	10	8
G+C (mol%)	57.01	57.01	57.13
tRNA	69	70	71
rRNA	5	5	4
CDS	4,509	4,498	4,362
Hypothetical CDS	946	937	847
PLFAM CDS	3,980	3,976	3,914
Coarse consistency	99.5	99.5	99.5
Fine consistency	98	97.9	98.2
CheckM completeness	100	100	100
CheckM contamination	0.2	0.2	0.2

**Fig. 1.**
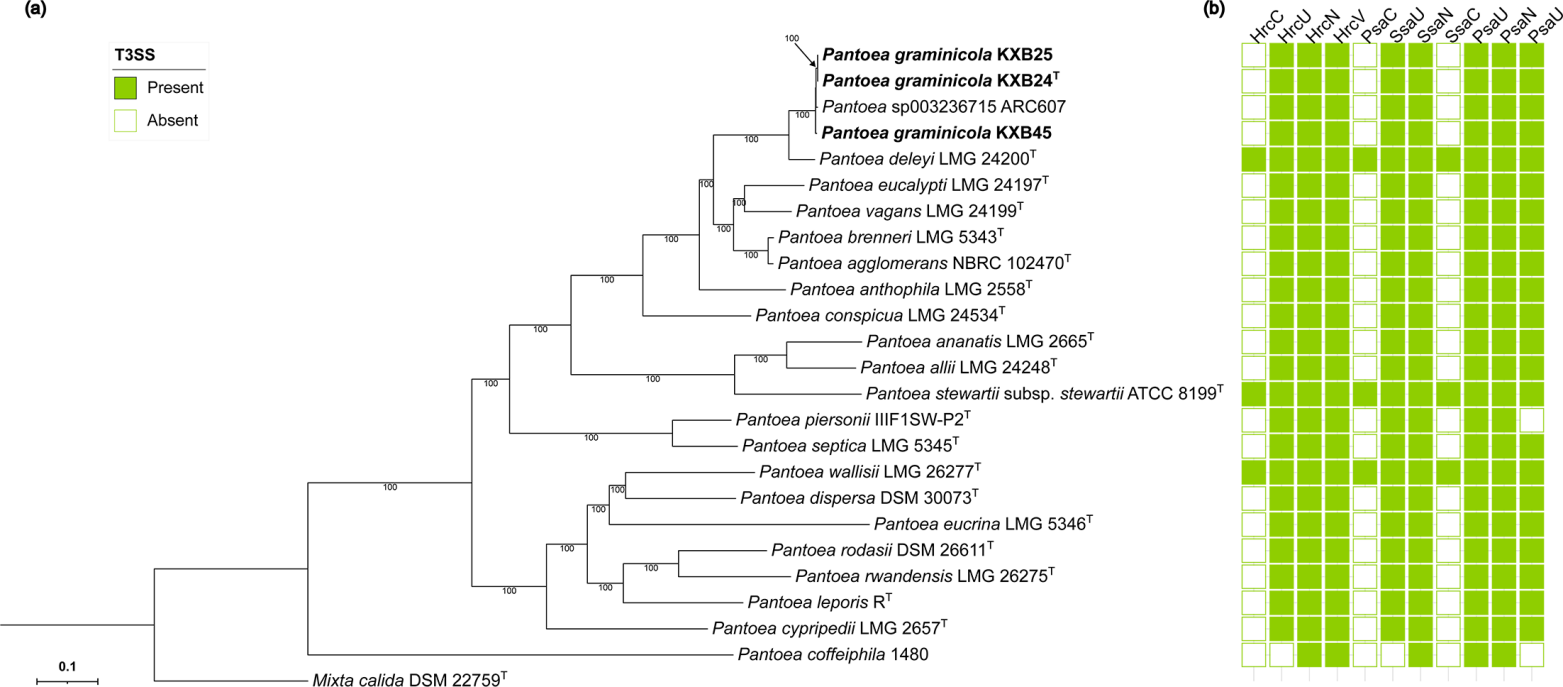
(**a**) Phylogenomic tree of *Pantoea* strains, including the isolates from this study (KXB24^T^, KXB25 and KXB45, in bold), and related species. The maximum likelihood tree is based on the alignment of 1,000 conserved genes across the genomes, constructed with RAxML. Bootstrap values greater than 70% are shown below the nodes. Type strains are indicated by a superscript ‘T’, and the tree is rooted with *Mixta calida* DSM 22759^T^. (**b**) Matrix showing the presence (coloured) and absence (white) of T3SS proteins across type strains of *Pantoea* spp.

To further evaluate the taxonomic placement of these strains, phylogenomic analyses were conducted. The BV-BRC phylogenomic analysis identified a total of 1,156 single-copy genes, of which 1,000 genes were used in the codon tree construction (Fig. 1a). Strains KXB24^T^, KXB25 and KXB45 consistently formed their own distinct clade separate from all recognized *Pantoea* species included in the analysis, with the exception of *Pantoea* sp003236715, which clustered with our strains. Among validly named type strains, their closest phylogenetic relative was *P. deleyi*. The TYGS analysis was unable to assign strains KXB24^T^, KXB25 and KXB45 as members of any species contained within its database. The resulting whole-genome phylogeny based on GBDP distances, including the novel strains and their closest known relatives, is presented in Fig. S2.

Strains KXB24^T^, KXB25 and KXB45 exhibited 99.35–100% ANI and 96.5–100% dDDH values (Table 1), surpassing the established species delineation thresholds of 95–96% for ANI and 70% for dDDH, indicating that these three strains represent the same species [29,34]. However, the ANI and dDDH values of the three strains in comparison with the other *Pantoea* type strains ranged between 74.99–94.26% and 20.5–57.9%, respectively, suggesting that these isolates belong to a novel species within the genus *Pantoea*. Notably, *Pantoea* sp003236715 shared 99.33% ANI and 96.4% dDDH with strain KXB24ᵀ, supporting its inclusion within the same species cluster.

We performed a comprehensive search for T3SS proteins across all 24 *Pantoea* spp. genomes (Table S1). The analysis revealed two distinct T3SS profiles among the species (Fig. 1b). The first pattern, exhibited by *P. deleyi*, *P. stewartii* and *P. wallisii*, included the presence of all 11 T3SS proteins. The second pattern, found in the majority of the strains, was characterized by the absence of three key proteins: HrrC, PsaC and SsaC. Notably, *P. coffeiphila* and *P. piersonii* exhibited a further modified T3SS profile, lacking additional proteins beyond these three. When comparing the newly described strains KXB24^T^, KXB25 and KXB45 to their closest relative, *P. deleyi*, we observed a distinct T3SS protein profile (Fig. 1b). Although the analysis was performed on draft genomes, the differences observed suggest possible evolutionary divergence in their secretion systems, which may have influenced adaptations in pathogenicity or host interactions relative to *P. deleyi*.

Upon comparison to the GTDB, isolates KXB24^T^, KXB25 and KXB45 were assigned to the taxonomy group *Pantoea* sp003236715, which contains three additional bacterial genomes belonging to strains previously described as members of the genus *Pantoea*, but which have not been assigned to any existing species. These isolates include the following: strain Ep11b (NCBI GenBank accession=GCA_040783975.1), which was isolated in 2022 from rice (*O. sativa*) leaves in Malaysia; strain S1C36_SP75 (GenBank accession=GCA_913774055.1), which was isolated in 2020 from the phyllosphere of rice plants in China and the genome of which was assembled from a metagenome; and strain ARC607 (GenBank accession=GCA_003236715.1) isolated in 2013 from rice seed in Benin. When compared to the representative genome of species cluster *Pantoea* sp003236715 (strain Ep11b, GCA_040783975.1), strains KXB24^T^, KXB25 and KXB45 produced ANI values of 99.38%, 99.40% and 99.48%, respectively, confirming that the Florida isolates and other members of the species cluster indeed belong to the same species. GTDB species clustering results show that *P. graminicola* sp. nov. has been isolated from multiple continents, suggesting a broad and potentially worldwide distribution. Additionally, the three other isolates contained within the species cluster had been found associated with rice plants, suggesting that the host range of the species includes both corn and rice. This finding is significant as corn and rice are often grown in rotation in the EAA region, and it is possible that *P. graminicola* sp. nov. could be moving between the two crops in this production system [35,36].

### Phenotypic characterization

The MALDI-TOF results indicated that the top species match for representative strain KXB24^T^ is *P. agglomerans* and *P. vagans* with scores of 1.96 and 1.71, respectively. The typical score range for probable species identification using MALDI-TOF is between 1.75 and 3.0. However, despite these scores, our ANI, dDDH and phylogenetic analyses clearly demonstrate that KXB24ᵀ is not the same species as *P. agglomerans* or *P. vagans*. This discrepancy highlights a known limitation of MALDI-TOF, which, while useful for rapid identification, may not always reliably distinguish between closely related species, particularly when their database references are incomplete or not sufficiently resolved.

The physiological and carbon utilization profiles of strains KXB24^T^, KXB25 and KXB45 were assessed using the Biolog Gen III MicroPlate system (Table S2). Biochemical profiling confirmed that all three strains belong to the genus *Pantoea*. Table 3 presents a summary of the phenotypic traits of strains KXB24^T^, KXB25 and KXB45, along with a comparative analysis against their closest phylogenetic relatives. In comparison with reference strains *P. deleyi* LMG 24200^T^, *P. agglomerans* CGMCC1.843^T^, *P. vagans* DSM 23078^T^, *P. allii* DSM 25133^T^ and *P. ananatis* ATCC 19321^T^, KXB24^T^ exhibited distinct utilization patterns for substrates such as d-maltose, α-d-lactose, gentiobiose, sucrose, acetoacetic acid, citric acid, γ-amino-butyric acid and sodium butyrate. While substrate utilization was generally similar between KXB24^T^, KXB25 and KXB45, certain substrates such as d-trehalose, d-turanose, d-mannose, *N*-acetyl-d-glucosamine, 3-methyl glucose, l-fucose, l-rhamnose, dextrin, Tween 40, d-serine, l-arginine, l-serine, acetic acid, d-malic acid, α-hydroxybutyric acid, nalidixic acid, lithium chloride and potassium tellurite were strain-specific. These phenotypic differences further support the distinctiveness of KXB24^T^, KXB25 and KXB45 from *P. deleyi*, *P. agglomerans*, *P. vagans*, *P. allii* and *P. ananatis*.

**Table 3.** Differential phenotypic characteristics of strains KXB24^T^, KXB25 and KXB45 and closely related strains in the genus *Pantoea*

Test	KXB24^T^	KXB25	KXB45	1	2	3	4	5
**Utilization of sugars**								
d-Maltose	w	w	w	+	+	−	−	−
d-Trehalose	+	+	w	+	+	+	+	+
d-Turanose	−	−	w	−	−	−	−	−
d-Mannose	w	w	+	+	+	+	+	+
α-d-Lactose	−	−	−	−	−	−	w	+
Gentiobiose	w	w	w	+	−	−	+	−
*N*-Acetyl-d-glucosamine	+	+	w	+	+	−	+	+
3-Methyl glucose	w	w	+	nd	+	nd	nd	nd
l-Fucose	−	−	w	−	−	−	w	−
l-Rhamnose	w	w	+	+	+	+	+	+
**Polymers**								
Dextrin	w	−	w	+	nd	−	+	+
Tween 40	−	−	+	+	−	−	−	−
**Amino acids**								
d-Serine	+	w	+	−	+	−	−	−
l-Arginine	w	w	+	nd	−	−	−	−
l-Serine	w	w	+	+	+	nd	nd	nd
**Carboxylic acids**								
Acetic acid	w	w	+	+	+	nd	nd	nd
Acetoacetic acid	w	w	w	nd	+	−	−	−
Citric acid	+	+	+	+	−	−	+	+
d-Malic acid	−	−	+	nd	+	nd	nd	nd
Formic acid	+	+	+	−	−	nd	nd	nd
α-Hydroxybutyric acid	w	w	−	−	+	nd	nd	nd
γ-Amino-butyric acid	+	+	+	+	+	−	−	−
**Others**								
Nalidixic acid	w	w	−	nd	+	−	−	−
Lithium chloride	w	w	+	nd	+	nd	nd	nd
Potassium tellurite	w	w	−	nd	+	−	−	−
Sodium butyrate	w	w	w	nd	+	−	−	−

1, *P. deleyi *LMG 24200T.

2,* P. agglomerans *CGMCC1.843T.

3, *P. vagans* DSM 23078T.

4, *P. allii* DSM 25133T.

5, *P. ananatis* ATCC 19321T.

+ refers to a positive reaction.

– refers to a negative reaction.

w refers to a weak reaction.

nd, not determined or not available.

**Fig. 2.**
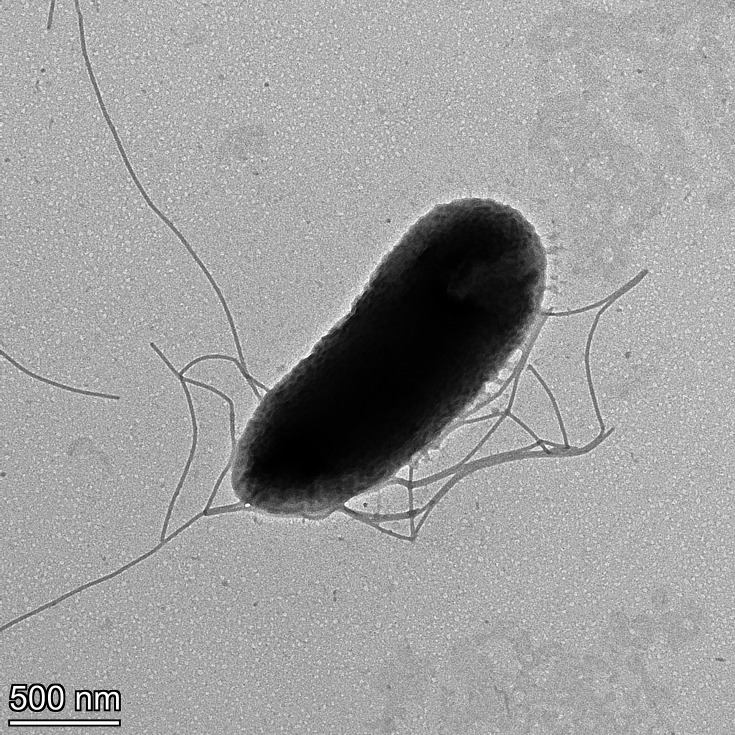
Transmission electron micrograph of *P. graminicola* sp. nov. strain KXB24ᵀ, showing rod-shaped cells with multiple peritrichous flagella.

Through transmission electron microscopy, cells of strain KXB24^T^ were revealed to be rod-shaped, ~1.8 µm in length and 0.73 µm wide, and to possess several peritrichous flagella (Fig. 2).

Based on polyphasic analysis that consists of genetic analyses including phylogenetic reconstruction based on core genes, ANI and dDDH and genome comparisons using the online TYGS server, along with MALDI-TOF MS analysis and biochemical profiling with the Biolog Gen III MicroPlate system, the three bacterial strains isolated from corn leaves in Florida represent a novel species within the genus *Pantoea*. We are proposing that these strains be placed in the new species, *P. graminicola*.

### Pathogenicity tests

Inoculations of strains KXB24^T^, KXB25 and KXB45 on corn resulted in inconsistent outcomes. While some leaves exhibited streaking, this was not observed consistently across all replicates. No visible symptoms or HRs were observed in rice, tomato or tobacco. However, re-isolations from both corn and rice successfully yielded colonies, which were confirmed through 16S rRNA sequencing. These results suggest that the strains can colonize maize and rice, but their ability to cause disease is not conclusive. In other pathosystems, *Pantoea* species have demonstrated intricate plant–pathogen interactions that are closely linked to the presence of specific plasmid-encoded virulence factors and biosynthetic gene clusters [35,36]. These findings suggest that pathogenicity in *Pantoea* is often context-dependent, shaped not only by genomic content but also by host specificity and environmental conditions. Our pathogenicity assays, while informative, may not have fully captured these dynamics. It is possible that under different host or environmental scenarios, or with alternative inoculation methods, the strains examined here could exhibit different pathogenic behaviours. Given these considerations, strains KXB24ᵀ, KXB25 and KXB45 represent interesting candidates for future studies aimed at elucidating the genetic and ecological factors that influence pathogenicity in *Pantoea*.

## Description of *Pantoea graminicola* sp. nov.

*Pantoea graminicola* sp. nov. (gra.mi.ni’co.la. L. neut. n. *gramen*, grass; L. masc./fem. n. suff. -*cola*, dweller; from L. masc./fem. n. *incola*, inhabitant, dweller; N.L. fem. n. *graminicola*, grass-dweller).

Colonies of *P. graminicola* sp. nov. on NA are circular, with smooth entire margins, convex, yellow and ~2.5 mm in diameter after 2 days of incubation at 28 °C under aerobic conditions. The colonies do not produce a diffusible fluorescent pigment when grown on King’s medium B. Cells are rod-shaped (~1.80 µm in length and 0.73 µm in width) with peritrichous flagella. The type strain does not induce a hypersensitive reaction in tobacco or tomato plants. All three strains, KXB24ᵀ, KXB25 and KXB45, were found to utilize d-cellobiose, d-fructose, d-galactose, α-d-glucose, sucrose, d-glucose-6-PO_4_, d-fructose-6-PO_4_, glycyl-l-proline, l-alanine, l-aspartic acid, l-glutamic acid, d-galacturonic acid, d-gluconic acid, d-saccharic acid, mucic acid, bromosuccinic acid, citric acid, formic acid, l-malic acid, l-lactic acid, γ-amino-butyric acid, sodium lactate, fusidic acid, guanidine HCl, tetrazolium violet and tetrazolium blue. They are negative for the utilization of d-raffinose, d-melibiose, α-d-lactose, stachyose, *N*-acetyl-d-galactosamine, d-aspartic acid, l-pyroglutamic acid, α-keto-glutaric acid, α-keto-butyric acid, β-hydroxy-d,l-butyric acid, propionic acid and sodium bromate. Additionally, the strains grow in environments with pH 5 and pH 6, and in the presence of 1%, 4% and 8% NaCl.

The DNA G+C content of the type strain is 57.01 mol%. The GenBank accession numbers for the 16S rRNA gene and draft genome sequences of strain KXB24^T^ (=NCPPB 4802^T^=LMG 33887^T^) are PV448656 and GCF_048452665, respectively.

## Supplementary material

10.1099/ijsem.0.006952 Uncited Supplementary Material 1.

10.1099/ijsem.0.006952 Uncited Supplementary Material 2.

## Abbreviations

ANI: average nucleotide identity
BV-BRC: Bacterial and Viral Bioinformatics Resource Center
c.f.u.: colony-forming unit
dDDH: digital DNA-DNA hibridization
dDDH: digital DNA–DNA hybridization
EAA: Everglades Agricultural Area
GBDP: Genome blast Distance Phylogeny
GTDB: Genome Taxonomy Database
HR: hypersensitive response
NA: nutrient agar
T3SS: type III secretion system
TYGS: Type (Strain) Genome Server
