# Supplementary material for: Pantoea graminicola sp. nov., a Gram-negative bacterium isolated from sweet corn (Zea mays L.) in the USA
Source: Int J Syst Evol Microbiol. 2025 Nov 3;75(11):006952. doi: 10.1099/ijsem.0.006952 (PMC12582532; doi:10.1099/ijsem.0.006952)
Supplement: Uncited Supplementary Material 1. [file ijsem-75-06952-s001.pdf]

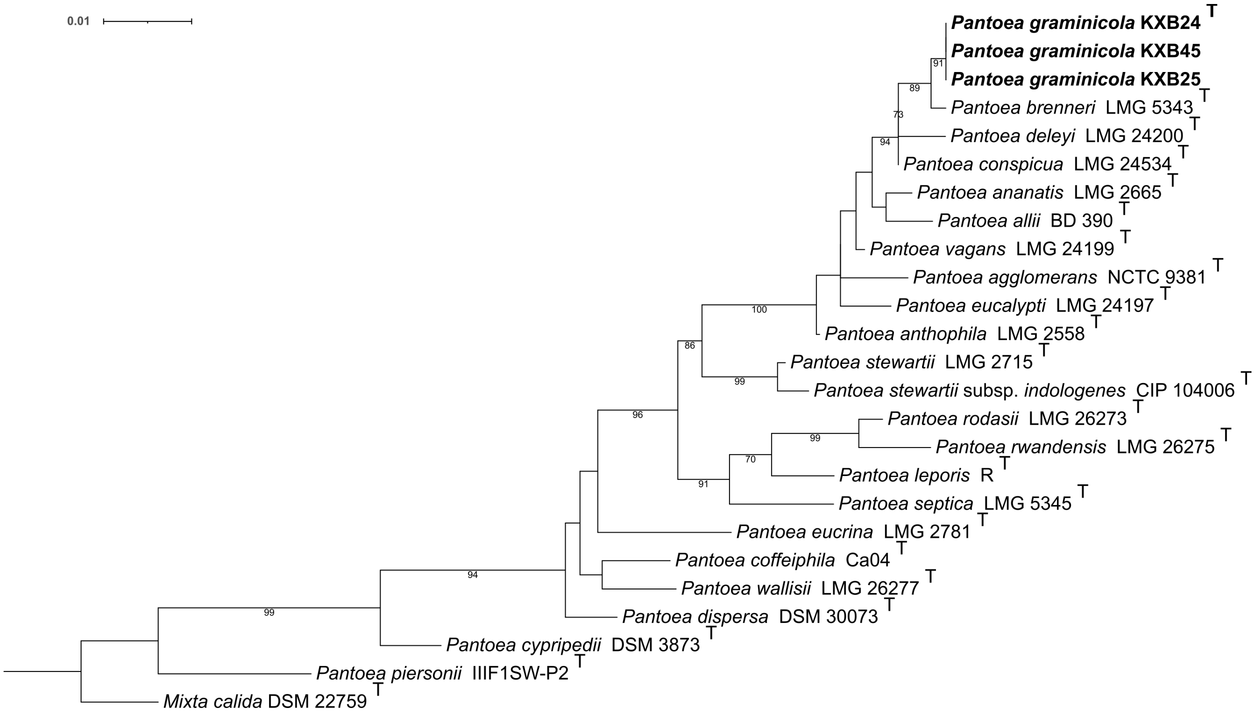

Figure S1. Maximum-likelihood tree showing the phylogenetic relationship of strains KXB24^{T}, KXB25, KXB45 (in bold) and type strains of all species within the *Pantoea* genus based on the aligned 1345 bp 16S rRNA gene sequences. The scale bar indicates 1% substitutions per site. Bootstrap values greater than 70% are displayed. Type strains are indicated by a superscript "T", and the tree is rooted with *Mixta calida* DSM 22759^{T}.

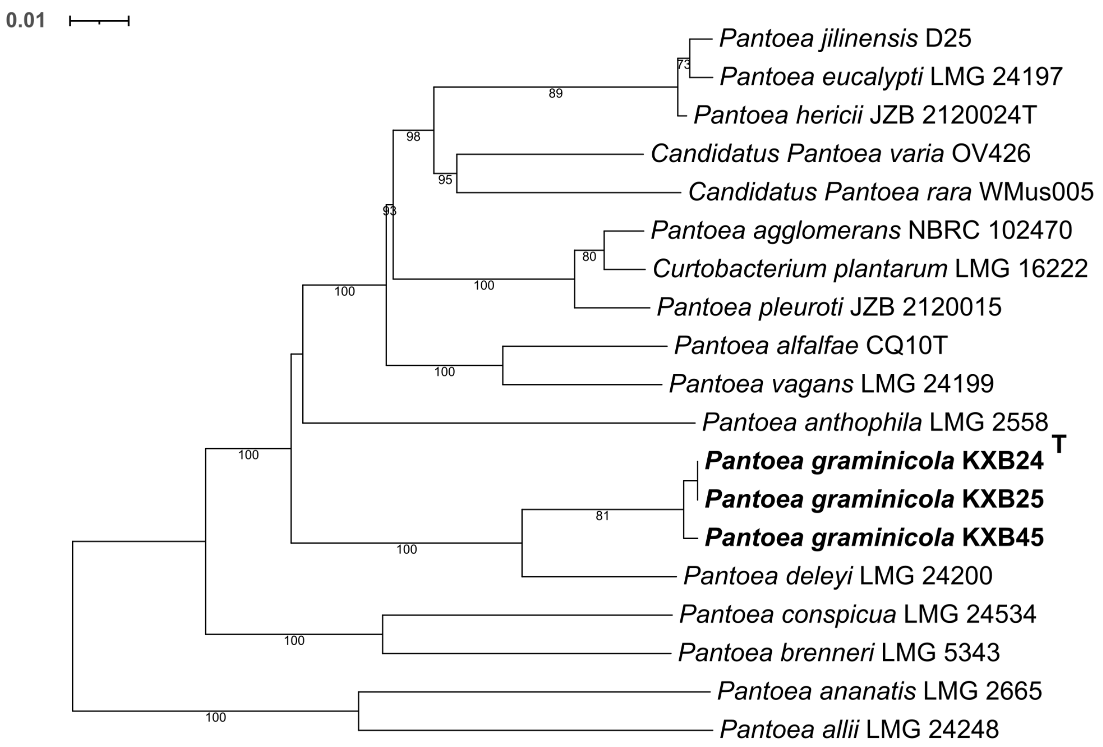

Figure S2. Tree inferred with FastME 2.1.6.1 from GBDP distances calculated from genome sequences. The branch lengths are scaled in terms of GBDP distance formula d5. The numbers above branches are GBDP pseudo-bootstrap support values > 60 % from 100 replications. The newly described strains KXB24^{T}, KXB25, and KXB45 are highlighted in bold.
